# Process evaluation of PsyCovidApp, a digital tool for mobile devices aimed at protecting the mental health of healthcare professionals during the COVID-19 pandemic: a mixed method study

**DOI:** 10.3389/fpsyg.2024.1378372

**Published:** 2024-03-21

**Authors:** Maria A. Fiol-deRoque, Maria J. Serrano-Ripoll, Sofia Mira-Martínez, Guadalupe Pastor-Moreno, Carolina Sitges, M. Esther García-Buades, Elena Gervilla, Mauro Garcia-Toro, Rocío Zamanillo-Campos, Ignacio Ricci-Cabello

**Affiliations:** ^1^Health Research Institute of the Balearic Islands (IdISBa), Palma, Spain; ^2^Primary Care Research Unit of Majorca, Palma, Spain; ^3^Research Network on Chronicity, Primary Care and Health Promotion (RICAPPS), Barcelona, Spain; ^4^Andalusian School of Public Health, Granada, Spain; ^5^Centro de Investigación Biomédica en Red de Epidemiología y Salud Pública, Instituto de Salud Carlos III, Madrid, Spain; ^6^Granada Biosanitary Research Institute, Granada, Spain; ^7^University of the Balearic Islands, Palma, Spain

**Keywords:** mixed methods, COVID-19, process evaluation, digital intervention, healthcare workers, mental health

## Abstract

**Introduction:**

PsyCovidApp, a digital intervention aimed at safeguarding the mental health of healthcare workers during the COVID-19 pandemic, demonstrated in a randomized clinical trial to yield significant improvements solely among healthcare workers undergoing psychotherapy or receiving psychotropic medication.

**Objectives:**

(1) To identify contextual factors and mechanisms of action that influenced the impact of PsyCovidApp during the aforementioned trial; (2) To pinpoint enhancements for optimizing its efficacy.

**Materials and methods:**

For the first objective, a process evaluation was conducted, amalgamating quantitative techniques (surveying 216 healthcare professionals who had utilized PsyCovidApp during the trial) and qualitative methods (in-depth interviews with 16 healthcare workers). The second objective involved a panel of seven experts, utilizing the RAND-UCLA methodology.

**Results:**

The quantitative study (response rate = 40%) revealed that 22% of respondents had not fully accessed the content of PsyCovidApp. The average usage time was 22.7 min/day, being higher (*p* < 0.05) among consumers of psychotropic medications. Contents related to relaxation and mindfulness were most highly rated. Acceptability and usefulness scores ranged between 7.3–7.5/10 points, with higher ratings (*p* < 0.05) among women and older healthcare workers. The qualitative study uncovered that the primary barriers to using PsyCovidApp were workload, lack of time, and exhaustion. Its primary mechanisms of action included emotion identification, mental health regulation (e.g., insomnia, intense emotions), and learning of techniques and skills. The expert panel reached a consensus on 29 proposals to optimize PsyCovidApp.

**Conclusion:**

The knowledge derived from this study could inform the design and implementation of future similar digital tools.

## Introduction

The healthcare crisis caused by the COVID-19 pandemic that began in 2020 caused an unprecedented increase in mental health issues among the healthcare professionals tasked with handling the crisis (Li et al., 2021; Canal-Rivero et al., 2023; Cruz-Ausejo et al., 2023), with high rates of acute stress (40%), anxiety (30%), burnout (28%), depression (24%), and PTSD (13%) (Serrano-Ripoll et al., 2020; Walton et al., 2020; Chirico et al., 2021; Saragih et al., 2021). Other long-term studies have also found a worsening of mental health 1 year after the pandemic (Viejo Casas et al., 2023).

In addressing the urgent need to identify effective resources that can be given to healthcare services to combat this problem, many systematic reviews highlight the lack of scientific evidence on the effectiveness of the interventions that were available up to that time, specifically designed for this population and context (Muller et al., 2020; Serrano-Ripoll et al., 2020; Drissi et al., 2021; Viejo Casas et al., 2023).

In June 2020, a team of psychologists and psychiatrists developed a digital intervention, in the form of a mobile phone app, called PsyCovidApp, designed expressly to safeguard the mental wellbeing of healthcare professionals with respect to the COVID-19 pandemic. PsyCovidApp takes a psycho-educational approach using cognitive-behavioral psychology (with content on handling emotions, lifestyle choice, workplace stress, burnout, and social support) and mindfulness. It also includes many practical tools (Serrano-Ripoll et al., 2021).

PsyCovidApp was studied in a randomized clinical trial (RCT) that started in June 2020. In total, 482 healthcare workers participated, the majority from around Spain’s Autonomous Communities (Fiol-DeRoque et al., 2021). After 2 weeks of follow-up, PsyCovidApp was not shown to significantly improve the overall mental health of participants. However, subgroup analyses do suggest significant improvements among healthcare professionals who took psychotropic medication or were receiving psychotherapy (Fiol-DeRoque et al., 2021). Upon completion of the RCT, 92% of participants (control and test groups) requested to have permanent access to PsyCovidApp, which suggests it has a high acceptance rate.

As has been suggested in previous systematic reviews (Kate et al., 2022; Delassalle and Cavaciuti, 2023), due to the scarcity of scientific evidence that exists on digital tools for handling mental wellbeing in healthcare professionals during pandemics (Witarto et al., 2022; Delassalle and Cavaciuti, 2023), we must look beyond the quantitative results of clinical trials and probe into the circumstances and mechanisms through which this kind of intervention does and does not produce its desired benefit. The objectives of the present study are as follows: first, to determine the impact of contextual factors, factors related to implementation, and the mechanisms of action that determine the effectiveness of PsyCovidApp, and second, to identify key aspects that can be included in PsyCovidApp to optimize its potential as a psychological support tool for healthcare workers facing similar crises in the future.

## Materials and methods

### Framework of reference

**Figure 1:**
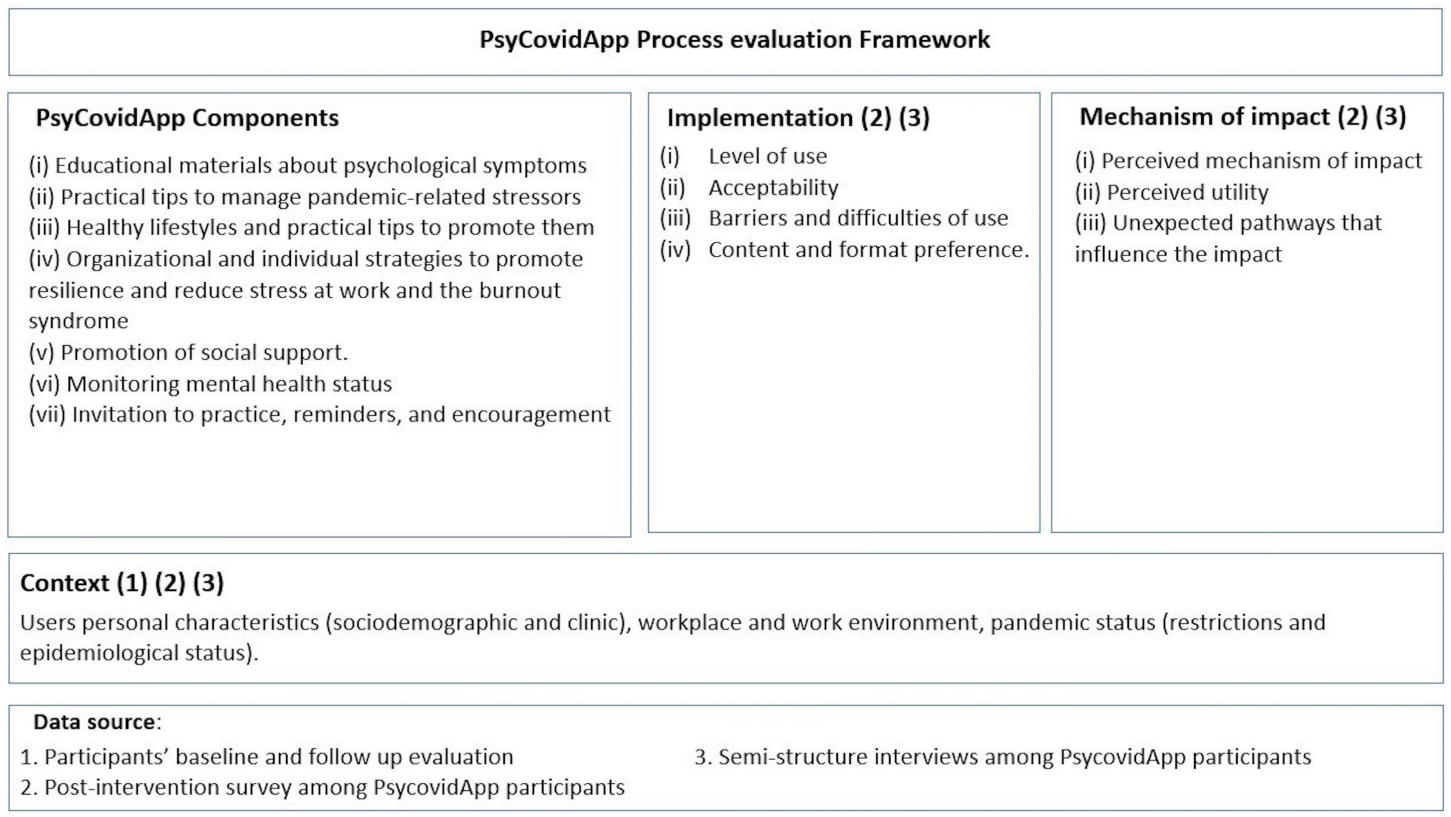
Framework of reference employed for conducting process evaluation. Adapted from Moore et al., 2015.

For our first objective, we carried out a process evaluation, following the guidelines of the Medical Research Council (Moore et al., 2015). This evaluation focused on three aspects: the context in which it took place, the form in which it was implemented, and the mechanisms through which the desired impacts were achieved (Figure 1). In carrying out the process evaluation, we took a mixed methods approach (Doyle et al., 2016), combining both quantitative and qualitative research techniques.

### Process evaluation of PsyCovidApp with quantitative techniques

We developed an *ad hoc* questionnaire (available in Appendix A) to gather information on participants’ experiences and use of PsyCovidApp during the RCT, i.e., usage of the tool over the 2 weeks of the RCT (7 items), usage of the tool after finishing the RCT (2 items), making use of the content on PsyCovidApp (15 items), and acceptability and perceived utility of PsyCovidApp (4 items). Sociodemographic, work-related, and clinical data were also gathered from participants.

All participants in the test group who authorized us to contact them regarding study updates once the RCT was finished were invited to complete an online questionnaire (*n* = 216/248), but the 234 participants form the control group were not invited, as they did not have access to PsyCovidApp during the RCT. After the first invitation, two reminders were sent. Responses to the questionnaires were recorded between December 22, 2020, and January 27, 2021 (6 months after the RCT was completed).

We performed a descriptive statistical analysis, calculating the number and percentage of participants in each category for the categorical variables, as well as the average and standard deviation for continuous variables. The differences between groups were analyzed by Chi-squared test for categorical variables and a comparison of means for continuous variables. We used parametric and non-parametric tests depending on the type of data. We analyzed the differences in scores on the questionnaire as a function of participant profile (age, sex, professional category, use of psychoactive medications, participation in psychotherapy, and changes on scales of depression, anxiety, and stress).

### Process evaluation of PsyCovidApp with qualitative techniques

We carried out a qualitative study using in-depth individual interviews to understand in detail the experiences of the professionals who used PsyCovidApp, exploring many possible barriers, suggesting improvements, and discussing the context in which it is used. We deliberately selected our sample to ensure representativeness among participants in terms of age, sex, professional category, how much they used the intervention, and whether or not they took medication and/or received psychotherapy. Interviews were performed over the telephone in January 2021, and they were recorded and transcribed. Five interviewers participated (MJS, GPM, EG, MEG, and CS); all of them have previous experience with qualitative methods. We made sure we performed the number of interviews required to answer our research question and ensure we had robust data. We created a topic guide (Box 1), which was agreed upon by the team of interviewers. The whole group met as a team and in pairs to train and standardize the interviews. Transcripts were analyzed using thematic analyses (Braun and Clarke, 2006), first individually, and then in pairs, for added rigor and to ensure that the data was seen from various perspectives. We used a topic guide as the departure point and then took an inductive approach to be able to identify emerging topics. One researcher (MJS) created an overview of the results, which was reviewed and validated by the rest of the team. The interviewers critically assessed their own role and the objective of the study so as to not influence the participants’ responses.

Box 1: **Topic guide used for individual qualitative interviews conducted as part of the process evaluation.**

**Acceptability, utility, usage, and difficulties encountered**

- Overall Impression: Broadly speaking, can you tell me about your experience participating in this project?
- Scope of the Intervention: How was accessing the intervention for you (how easy or difficult did you find it)? Were you able to access the intervention via your mobile phone? Describe the process...
- Dosage (Extent of Usage): Do you recall how frequently you used it? (a lot, quite a bit, sparingly, daily, whenever possible...). Share your usage experience.
- Timing/Context: Do you remember at what times of day it was better or more favorable for you to access the App? In what context did you use the application (home, work, etc.)?
- Action Mechanisms: To what extent has this App been useful in caring for your mental health during this pandemic period? If it has been useful, how do you think the App has aided you?
- Preferences: Which contents did you like the most? Which ones are the least?
- Usability: How easy or difficult did you find the App to use?
- Utility: How useful have you found the contents?
- Level of Acquired Knowledge: How much do you think you have learned from these contents?
- Behavioral Changes: To what extent have you applied what you have learned in your day-to-day life?
- Regarding Timely Messages: What can you tell us about their frequency, relevance, content, etc.?

**Barriers**

What barriers do you think people might face in using the App?

**Suggestions for improvement**

Did you feel anything was missing in the contents? Is there any content you would eliminate or reinforce (e.g., audiovisual vs. text, for instance)?
What do you think we could improve to make this App more useful?
What do you think we could enhance to make this App more appealing?
What do you think we could improve to make this App easier to use?
Would you suggest any improvements for the intervention for widespread, large-scale usage?

### Panels of experts for optimizing PsyCovidApp

For our second objective (to identify possible points of improvement for optimizing the impact of PsyCovidApp), we consulted a panel of experts, which was put together following the RAND-UCLA methodology (Fitch et al., 2001). A total of seven experts with multidisciplinary backgrounds were recruited, including specialists in mental health, lifestyle, digital health, and app design. Before participating on the panel, they received information on PsyCovidApp (i.e., the scientific article on the results of the RCT (Fiol-DeRoque et al., 2021), and the overview of the quantitative and qualitative process evaluation).

We performed two rounds of queries, both digital, via online questionnaires. In the first round, all possible improvements along with their rationale were presented, and the panel was asked to classify these suggestions into the preestablished categories posed by the team of researchers, though they were allowed to add additional categories if necessary. The suggestions received were then compiled, recategorized, and duplicates were eliminated. The second round involved asking the panel to individually assess the suggestions, independent of all others. The panel then considered the suggestions from two points of view: perceived usefulness (Up to what point is it likely that this suggestion will significantly improve PsyCovidApp?) and feasibility (Up to what point is it feasible to carry out this measure?). Both aspects were evaluated on a visual, analog scale from 1 to 9.

For each suggestion for improvement, we determined its level of pertinence and appropriateness and the level of agreement reached among the experts. Pertinence and appropriateness were classified as follows: pertinent/appropriate (unanimously ranked 7–9), unclear (with a median of 4–6, or any disagreement), or not pertinent/inappropriate (unanimously ranked 1–3). We assessed agreement among the experts individually for each of the suggested improvements, considering there to be a consensus if 90% of the experts ranked the suggestion in the same range (1–3, 4–6, and 7–9) (Fitch et al., 2001).

## Results

### Process evaluation

#### Quantitative analysis

Of the 216 healthcare professionals invited to complete the online questionnaire, 87 responded (a response rate of 40.3%). This sample was representative of the participants in the RCT intervention group, with no statistically significant differences in any of the clinical or sociodemographic variables reported (Appendix B), except for age (the group of participants in the RCT was significantly younger).

Of the 87 healthcare professionals who participated, 89.7% were women (*n* = 78) with an average age of 45.4 years old (SD: 10.1 years) and an average professional career of 19.2 years (SD: 8.8). By professional category, 35.6% (*n* = 31) were nursing assistants, 33.3% (*n* = 26) were doctors, and 29.9% (*n* = 26) were registered nurses. Regarding their clinical profiles (established during the base assessment of the RCT), 18.4% (*n* = 16) of the healthcare professionals claimed to take psychoactive medications, and 9.2% (*n* = 8) received psychotherapy before participating in the RCT. The average score on the DASS-21 scale was 6/21 (SD: 4.0).

Most of those interviewed (85%, *n* = 74) stated that they had no technical issues when accessing PsyCovidApp, and 78% (*n* = 65) claimed to have accessed all the content on the app (Table 1). The average age [SD] of the participants that accessed all the content was greater than the average age of those who left some modules unseen (*p* = 0.006): 46.96 [9.45] versus 39.27 [9.97] years old, respectively.

**Table 1:** PsyCovidApp usage by healthcare professionals assigned to the intervention group of the randomized clinical trial.

	*N* (total = 87)	%
**Technical issues in accessing PsyCovidApp**		
No	74	85.06
Yes	8	9.20
I do not know/I do not remember	5	5.75
**Access to all content**		
Yes	68	78.16
No	15	17.24
I do not know/I do not remember	4	4.60
Reasons for not accessing all content (**N** = 15)*		
Lack of time	7	46.67
Lack of interest	7	46.67
Previous knowledge on the topic	7	46.67
Content deemed irrelevant	4	26.67
Access problems with the App	1	6.67
Others	1	6.67
**PsyCovidApp use first week**		
1 day	4	4.60
2–3 days	19	21.84
4–6 days	34	39.08
All 7 days a week	23	26.44
Did not access at all	3	3.45
I do not know/I do not remember	4	4.60
**PsyCovidApp use second week**		
1 day	8	9.20
2–3 days	23	26.44
4–6 days	28	32.18
All 7 days a week	15	17.24
Did not access at all	5	5.75
I do not know/I do not remember	8	9.20
**Daily use of PsyCovidApp (minutes)**		
Mean (SD)	22.7 (20.6)	
Median (IQR; range)	15 (10–30; 0–120)	
0 min	2	2.30
1 to 5 min	11	12.64
6 to 15 min	33	37.93
16 to 30 min	31	35.63
31 to 60 min	3	3.45
> 60 min	7	8.05
**Weekly use of PsyCovidApp (hours)**		
Mean (SD)	5.66 (4.85)	
Median (IQR; range)	4 (2–8; 0–24)	
0	2	2.30
1–2	23	26.44
3–5	23	26.44
6–10	23	26.44
> 10	8	9.20
NA	8	9.20

IQR, interquartile range; *N*, number of participants; SD, standard deviation. *non-exclusive responses.

Around half (46.7%) of the people who claimed that they had not accessed all the content gave reasons that include a lack of time, a lack of interest, and a lack of relevance (as they already had previous knowledge in the area).

Regarding the amount of time, they used PsyCovidApp during the RCT (Appendix C), 47% stated they used the app more than 4 days a week during the 2 weeks of the trial, with slightly more use during the first week, 65.5% (n = 57), than during the second week, 49.4% (*n* = 43). The time that participants dedicated to using the app was 22.7 min/day (SD: 20.6; median: 15, IQR: 10–30). Regarding total access time over the week, on average, participants spent 5.66 h/week on the app (SD: 4.85; median: 15, IQR: 10–30). Additionally, those that took psychotropic medication showed a higher average usage time (*p* < 0.05) than the rest of the participants (24.4 versus 22.3 min).

About the four dimensions assessed for acceptability and perceived utility of PsyCovidApp, average scores oscillated between 7.32 and 7.49 points out of 10 (Table 2).

**Table 2:** Assessment of Acceptability and Perceived Utility of PsyCovidApp Ғ Ғ the data represent the average score (SD) of the four scales (theoretical range between 1 and 9 points in all of them); *p* = level of statistical significance.

	Appropriate tool	Quantity of information	Utility of the tool	Likelihood to recommend PsyCovidApp
Sex	*p* = 0.190	p = 0.006	*p* = 0.217	*p* = 0.174
Man (*n* = 9)	6.33 (2.55)	5.78 (2.59)	6.22 (2.91)	6.44 (2.74)
Women (*n* = 78)	7.43 (2.17)	7.95 (1.88)	7.46 (2.28)	7.61 (2.34)
Age	*p* = 0.019	*p* = 0.005	*p* = 0.012	*p* = 0.008
<36 years (*N* = 17)	5.88 (2.91)	6.47 (2.45)	5.88 (3.18)	5.88 (3.20)
36–45 years (*N* = 25)	7.60 (1.96)	7.72 (2.11)	7.44 (2.18)	7.56 (2.16)
46–55 years (*N* = 29)	7.38 (1.86)	7.62 (1.88)	7.45 (1.78)	7.66 (1.84)
>55 years (*N* = 29)	8.31 (1.71)	9.06 (0.93)	8.5 (1.93)	8.81 (1.80)
Total *(n = 87)*	7.32 (2.22)	7.72 (2.06)	7.33 (2.36)	7.49 (2.39)

When analyzing the assessments made by participants broken down by profile, we see certain differences related to sex and age. Women scored the quantity of information contained on PsyCovidApp higher than men, and older participants gave higher scores on metrics concerning the appropriateness of the tool, the quantity of information, the usefulness of the tool, and the probability of recommending PsyCovidApp. No differences were noted with regard to professional category, taking psychoactive medications, participating in psychotherapy, or a change in DASS-21 score (data not shown).

### Qualitative analysis

Sixteen women and three men were interviewed (six nurses, six doctors, and seven nursing assistants from different fields), aged between 29 and 61 years old. In Box 2 we show selected illustrative quotes in text form.

Box 2: **Examples of verbatim quotes from healthcare professionals (*n* = 16) who participated in the qualitative interviews.**

1. General acceptance of the app

“It seemed very interesting to me, and I found it very helpful. It really opened up for me... Many times, we have information, I do not know how to explain it, that we, as healthcare professionals, do not apply to ourselves. I mean, we know what anxiety is, we know what stress is, but sometimes, we put it aside in our minds. It was very good to remember things, to recall anxiety, to remember the symptoms of seeing them in myself and be able to act upon them. It was very beneficial for me, very beneficial.” (woman, 46 years old, pediatric nurse)

1. App utility

“Yes, it was very helpful for me. It was great for me. I do not know if maybe, at that moment, I was alone, and it helped me see things in a different way, I do not know, but it really helped me a lot... I think, to make me stronger, for example, to know how to get out of situations. A bit more like that because there are times when I say, I see people who are worse off than me. Because with everything going on, I do not even know how we are managing to cope.” (woman, 40 years old, healthcare assistant in polyvalent care)

1. App usage (dose–response)

“When I received your notifications. The app would send you a notification for one reason or another, and then I would use it. If there was no notification, I would not use it. Always in the morning, first thing.” (man, 60 years old, hospital doctor)

1. Barriers and difficulties encountered in using the app

“One would be what I told you, that we always have time for other things except for taking care of ourselves. This could be one, I mean, it’s hard for us. Like I said before: ‘I know what I have to do. I do not do it, but I know that...’ This would be one. Yes, because, besides, you know what happens? We are under a lot of tension, we really need it, but since we are so overwhelmed, when we leave here, we dedicate a little time to the family and then to other things we have to do, and then you say, ‘Well, I’ll do it tomorrow.’ I think this would be one thing. I could not tell you more.” (woman, 61 years old, primary care doctor)

1. Content preferences

“The ones I liked the most were the calming topics, those were about breathing, exercises... I do not remember very well what that section was called, but there were breathing exercises, concentration exercises, relaxation. Those exercises are what I liked the most.” (man, 55 years old, internal medicine doctor)

1. Level of knowledge acquired and/or applied

“Well, I’ve learned, I think I would say, I do not know how to explain it. I have not learned to make life different, I’ve learned more to live with myself. I do not know if this explains it. [...] From zero to 10, I would say I’ve learned? A 9. Because in that period of March, April, May, or June when this caught us by surprise, there were moments when I felt a bit lost. I did not even know how to face it but how to assimilate certain things that were new and yes, in those months, it was good.” (woman, 61 years old, healthcare assistant in Internal Medicine)

1. Temporal messages (frequency, relevance, content, etc.)

“For me, yes, because it was like forcing myself a bit. The reminder would arrive, and I’d say: ‘Okay, I’ll take a look.’ For me, it was an important part at that moment when you had 8,500 things in your head, and it was like a really good moment in the morning because, I tell you, it coincided more or less with the time I was having breakfast, and it suited me.” (woman, 41 years old, care assistant in a care center)

1. Improvement suggestions

“No, improve? I think it’s very good. There are always things, but if I were to review everything and analyze it, this yes, but right now, nothing comes to mind... It’s very well expressed, the contents are very good, and all that I’ve worked on, I truly find it very good, it’s adapted and very good.” (woman, 51 years old, assistant in an Infectious Diseases Unit)

1. Formats

“I liked the audiovisuals. In fact, I think it was part of what I also used at night. Now it’s been a few days since I used them, but I remember they were like this because just listening to the voice always relaxes a lot. Yes, the fact that they were explaining it and as I did it at night, it also rested my eyes. That format, I liked it, very well.” (woman, 46 years old, primary care doctor)

Regarding the perceived acceptability and usefulness of PsyCovidApp, participants accepted and positively evaluated the tool, which was perceived to be useful considering the critical situation that was playing out, which itself was marked by excessive work, exhaustion, and stress. Participants highlighted the usefulness of the tool in raising awareness of their own psychological state. For some participants, PsyCovidApp served as an adjuvant to psychoactive medication or psychotherapy, while others, though not receiving treatment, found the app to be a support, helping them to sleep better, for example. A minority of users rated the tool not useful, finding the resources provided to be too basic.

The main barrier to the use of PsyCovidApp was a lack of time. PsyCovidApp was used mainly outside of the workplace, given the difficulty professionals had in using it at work (because of personal protective equipment and an excessive workload). The process of downloading and using the app was perceived to be simple. Some participants expressed that the organization of the contents into hierarchical modules and submodules made it more difficult to use.

Daily notifications were perceived to be useful for reminding participants to keep using PsyCovidApp. However, some users suggested that being able to define the time of the notification would be helpful, as they were notified too early, and subsequently forgot to use the app, making the notification useless. The contents that were most well-received were those related to breathing, relaxation, and meditation, as well as tools to deal with sleep problems. On the other hand, contents on healthy lifestyles were less attractive, and they were found not to provide new information or practices. In general, participants felt that they acquired practical abilities (e.g., around half of the participants stated that they learned something and had made use of suggested relaxation techniques), but that they got less theoretical knowledge.

Suggestions were made to improve the tool’s level of personalization and interaction. It was also suggested that the app provide some of the content in greater depth and offer final self-assessment questions. Another suggestion involved being able to receive feedback on the user’s mental health or having professional assistance available at some point (e.g., via chat). Another suggestion was to improve the look and design of the app and incorporate some gamification.

### Expert panel

A panel consisting of seven experts (see acknowledgments) actively participated in two rounds of identifying and prioritizing improvement proposals. In the first round (identification), a total of 57 proposals were made (Appendix D). In the second round (prioritization), 29 (51%) of these proposals were agreed upon as highly useful and feasible. These proposals are shown in (Box 3). Of the remaining proposals, nine (16%) were perceived as useful but not feasible, 13 (23%) as feasible but not useful, and six (11%) were considered neither useful nor feasible.

Box 3: **Suggested improvements for PsyCovidApp with a high level of consensus on their utility and feasibility.**

Utility

- Add a podcast format for the same written text
- Deepen the techniques and strategies aimed at protecting the mental health of healthcare professionals
- Prioritize the App’s functions concerning the primary need it aims to address

Atractiveness

- Improve visual aspects. The UI (User Interface) involves neuroscience: Use more vibrant colors, rounded buttons, calls to action, enriching images, highlighted texts
- Ability to change font size
- Summary of completed sections or modules
- Add (unlock) contents based on the completion of objectives/themes
- The information is very theoretical, alter the language used. The positive aspect is that it can be applied by professionals in their routine clinical practice with other users

Easy of use

- Provide a content outline
- Enable access to modules directly from the content guide
- Modify the navigation with “BACK and NEXT” buttons to direct buttons. Add an easily accessible exit to the main menu without alarmist indications, but more user-friendly and empathetic
- Direct attention and facilitate decision-making
- Modify multimedia content or the system so that (1) the mobile device does not lock after a few seconds; (2) multimedia content (e.g., audio recordings) continues to play
- Add a reminder system or mode for the last completed module/section/theme; have a view from the index showing how many steps are yet to be seen or have been seen in each module. For example: Mark different modules already consulted with a different color/tonality
- Reduce the amount of information and redesign UI (User Interface) pages to make functionality more user-friendly and to better connect with users

Widespread large scale use

- Adapt content to a “non-COVID” context
- Specify where individuals can seek help (mental health/physical activity/nutrition)
- Resilience is explained in one of the modules but appears in the module introduction as if it were a well-known concept

Adding contents

- Offer different difficulty levels: create modules on the same topic but with more advanced content
- Include some simpler content, especially in emotional regulation
- Possibility to include some scales (anxiety, Maslach Burnout Inventory...)
- Add self-assessment forms or systems
- Add explanations or clarification for some technical terms used. For example: verborhea
- In modules like possible stress reactions, it might be interesting for the user to mark the identified reactions as a self-awareness process
- Add a module on pain
- Add notifications at specific times of the day

Removing contents

- Modules M2.1 and M2.2 seem somewhat redundant, perhaps consolidating them into one
- Remove and update invalid links (e.g., the link from the Ministry of Health, Consumption, and Social Welfare in module 2.2 of section 11/13 in “Practical ideas for physical exercise”)

Feedback

- Automatic (and if possible, personalized) feedback

## Discussion

With our process evaluation we are able to provide more in-depth knowledge on the contextual factors, implementation factors, and action mechanisms that determined the effectiveness of PsyCovidApp. Despite the app being considered a useful and acceptable tool, its level of use varied, and was higher among healthcare professionals who were already taking psychotropic medications or receiving psychotherapy. The main obstacles to implementation were based on the individual and the context, e.g., being overworked, lacking time, or being exhausted. The main action mechanisms that contributed to improving mental health were related to aiding a participant in identifying his/her own emotions, regulating different aspects of mental health (specifically insomnia and having intense emotions), and promoting the learning of abilities and techniques (e.g., relaxation and mindfulness). The panel of experts identified 29 suggestions about which a high level of consensus on their feasibility and usefulness in optimizing the beneficial effects of PsyCovidApp.

### Strengths and limitations

The main strength of this study is its use of mixed methods, which has allowed us to obtain data from various sources and analyze them using a variety of methodologies. An important strength of the qualitative study is that it complies with all the main criteria of methodological rigor: credibility, reliability, transferability, and confirmability (Steinke, 2004).

Our study is not free from limitations. First, although we invited all the participants of the test group from the RCT to complete the quantitative questionnaire, the response rate was 40%. Although we do not see any significant differences in the clinical or sociodemographic characteristics of participants and non-participants, it is possible that those who participated were specifically those who had a more favorable experience with or opinion of PsyCovidApp. This possible participation bias could mean that we are overcounting the high ratings we found for perceived appropriateness and usefulness. Secondly, information on the use of the application and the contents that were consulted was gathered 6 months after the RCT was completed. Although most (92%) participants requested to have PsyCovidApp reactivated so that they could continue to use it after the RCT, we cannot discard the possibility that recall bias may have affected their answers. Finally, it is worth noting that our evaluation of the implementation of PsyCovidApp is limited by the fact that it was not possible to collect actual usage data from the application during the RCT. Thus, usage data is based on self-referenced information provided by the participants themselves.

### Our results compared to previous literature

As suggested in a recent systematic review, the scientific evidence on digital interventions aimed at protecting the mental health of healthcare professionals that were on the front lines of the COVID-19 pandemic is scarce, due largely to the scant number of studies that have been carried out (Drissi et al., 2021). There is a greater amount of evidence on digital interventions aimed at improving mental health for the general population during the COVID-19 pandemic (Witarto et al., 2022; Ye et al., 2022). Findings from these studies suggest that such interventions could potentially be effective at improving symptoms of anxiety, depression, stress, and insomnia during a pandemic. Although there is evidence available to suggest that interventions like PsyCovidApp–based on cognitive-behavioral therapy and mindfulness–are effective, adherence and level of use tend to be lower in digital interventions than interventions led by a professional, which conditions the overall effectiveness of these kinds of interventions.

The quantitative assessment of our process evaluation revealed a greater level of use of PsyCovidApp among participants who were taking psychoactive medications, while the RCT showed mental health improvements in the subgroup of those taking such medications or those receiving psychotherapy (Fiol-DeRoque et al., 2021). This could be indicative of a dose–response effect. However, it might also suggest a greater effectiveness of the app among people with more serious mental health problems, which would be in line with the results of a recent meta-analysis (Serrano-Ripoll et al., 2022) suggesting that mental health apps are more effective in patients with more serious conditions.

Our results are consistent with those presented in a recent systematic review, which identified four factors that determine the continued use of digital tools for dealing with mental health: app personalization, the use of reminders, a “friendly” design and being technically stable (making them easier to use), and complementary support from a person (Jakob et al., 2022). These four factors were identified both in the results of our qualitative study as well as by the panel of experts.

Being able to combine PsyCovidApp with personal psychological support was identified as beneficial both in the qualitative interviews and by the panel of experts. To this end, similar support can be found in a recent systematic review, which concludes that including the support of a mental health professional improves the effectiveness of and adherence to mobile phone apps designed to improve mental health (Szinay et al., 2020).

With regard to daily notifications, previous studies have provided evidence that these reminders can have a negative effect if they are received at times or places where the use of such apps would be inappropriate, as they deal with sensitive or stigmatized topics, i.e., mental health (Szinay et al., 2020). Being able to personalize the notifications by defining a timetable and frequency could help improve the level of use of PsyCovidApp by healthcare professionals.

### Relevance of the results to clinical practice and research

The results of our study provide valuable information for the design, development, and implementation of future applications destined to provide mental health support to healthcare professionals facing crises, in addition to applications destined to provide general mental health support and support for other chronic diseases.

Our results reveal the need for healthcare professionals to have time to access the resources offered to them to improve their mental health. Simply offering the tools is not enough. Rather, there must be a workplace environment that allows them to make use of the tools as part of their job.

## Conclusion

Our study of a process evaluation has allowed us to create information on the acceptability, level of use, mechanisms of impact, and context of the PsyCovidApp intervention. The results obtained, along with the 29 suggestions identified by the panel of experts, represent new scientific evidence upon which to base future efforts aimed at optimizing the implementation and effectiveness of PsyCovidApp, and upon which to structure the design and development of new tools.

## Data availability statement

The raw data supporting the conclusions of this article will be made available by the authors, without undue reservation.

## Ethics statement

The studies involving humans were approved by Research Ethics Committee of the Balearic Islands (IB 4216/20). The studies were conducted in accordance with the local legislation and institutional requirements. The participants provided their written informed consent to participate in this study.

## Author contributions

MF-R: Conceptualization, Formal analysis, Investigation, Methodology, Validation, Writing – original draft, Writing – review & editing. MS-R: Conceptualization, Formal analysis, Investigation, Methodology, Validation, Writing – original draft, Writing – review & editing. SM-M: Formal analysis, Methodology, Writing – original draft, Writing – review & editing. GP-M: Formal analysis, Methodology, Validation, Writing – original draft, Writing – review & editing. CS: Formal analysis, Methodology, Validation, Writing – original draft, Writing – review & editing. MG-B: Formal analysis, Methodology, Validation, Writing – original draft, Writing – review & editing. EG: Formal analysis, Validation, Writing – original draft, Writing – review & editing. MG-T: Funding acquisition, Resources, Visualization, Writing – original draft, Writing – review & editing. RZ-C: Project administration, Validation, Writing – original draft, Writing – review & editing. IR-C: Conceptualization, Funding acquisition, Validation, Writing – original draft, Writing – review & editing.
