# Supplementary material for: Process evaluation of PsyCovidApp, a digital tool for mobile devices aimed at protecting the mental health of healthcare professionals during the COVID-19 pandemic: a mixed method study
Source: Front Psychol. 2024 Mar 21;15:1378372. doi: 10.3389/fpsyg.2024.1378372 (PMC10994142; doi:10.3389/fpsyg.2024.1378372)
Supplement: Supplementary file 1 [file Data_Sheet_1.PDF]

MULTIMEDIA APPENDIX 1. Questionnaire used to assess PsycovidApp

1- GENERAL CHARACTERISTICS OF PARTICIPANTS (Sociodemographic, occupational, and clinical data)

- Age (years)
- Gender
  - Male
  - Female
  - Others
- Professional category
  - Doctor/Resident doctor
  - Nurse/Resident nurse
  - Auxiliary Nurse Care Technician
  - Others
- Years of professional experience (including residency years)
- Medical specialty
- Workplace during the COVID-19 pandemic
- Hospital: outpatient clinics
  - Hospitalization: admitted patients
  - ICU
  - Primary care
  - Nursing homes
  - Home care
  - Operating rooms
  - Emergency rooms
  - Others [open-ended response]
- How often do you use mobile applications (Apps)?
  - Never
  - Once a year
  - Once a month
  - Once a week
  - Daily
- How many Apps do you usually use?
  - None
  - 1 or 2
  - Between 3 and 5
  - Between 6 and 10
  - More than 10

2- USE OF PSYCOVIDAPP (PsycovidApp-Clinicovery: use after the intervention and use during the intervention)

- 2.A: After completing the clinical trial, have you continued using the App? If yes, indicate the approximate time you have dedicated to it since then.
  - No
  - Yes, I have used it for less than a week
  - Yes, I have used it between 1 and 2 weeks

    - Yes, I have used it between 2 and 4 weeks
    - Yes, I have used it between 1 and 2 months
    - Yes, I have used it for more than 2 months
    - I don't know / I don't remember
- 2B: Have you used PsycovidApp in the last fourteen days?
    - Yes
    - No
- 2C: Did you experience any technological problems that prevented you from using the App correctly during the study?
    - Yes
    - No
    - I don't know / I don't remember
- 2D: Did you access all the content/modules of the App?
    - Yes
    - No
    - I don't know / I don't remember
- 2E: If you couldn't access all the content, do you remember why? Check all that apply:
    - Lack of time
    - Lack of interest
    - Problems accessing the App
    - Problems downloading content from any module
    - I already had prior knowledge of some modules and didn’t consult them
    - Some content seemed irrelevant to me, so I didn't consult it
    - I don't know / I don't remember
    - Others [open-ended response]
- 2F: During the FIRST week you had access to the App, do you remember how many days you accessed it?
    - All seven days of the week
    - 4-6 days
    - 2-3 days
    - 1 day
    - I didn’t access it at all
    - I don't know / I don't remember
- 2G: During the SECOND week you had access to the App, do you remember how many days you accessed it?
    - All seven days of the week
    - 4-6 days
    - 2-3 days
    - 1 day
    - I didn’t access it at all
    - I don't know / I don't remember
- 2H: Approximately, how much time (in MINUTES) do you think you spent on average PER DAY consulting the App during the clinical trial? For example: 5, 30, 120, ...
- 2I: Approximately, how much time (in HOURS) do you think you spent in TOTAL consulting the App during the 14 days of the clinical trial?

3. IMPACT MECHANISM (PsycovidApp-Clinicovery: intervention content).

- MODULE 1. EMOTIONAL TRAINING [Why are emotions good? For example: excessive worry, irritability, stress, anxiety, etc.]
- MODULE 1. EMOTIONAL TRAINING [Awareness and mindfulness]
- MODULE 1. EMOTIONAL TRAINING [Practical breathing exercises]
- MODULE 1. EMOTIONAL TRAINING [Progressive relaxation exercises by Jacobson]
- MODULE 1. EMOTIONAL TRAINING [Practical imagination-based relaxation exercises]
- MODULE 1. EMOTIONAL TRAINING [Emotional regulation skills: excessive worry, controlling irritability, recommendations for addressing stress or anxiety, guidelines for dealing with fear and improving mood.]
- MODULE 1. EMOTIONAL TRAINING [Skills for surviving emotional crises (distracting oneself with different strategies, providing oneself with positive stimuli and relaxing senses, improving the current moment through our mind, weighing pros and cons)]
- MODULE 2. HEALTHY LIFESTYLES [Physical activity. Practical ideas for exercising]
- MODULE 2. HEALTHY LIFESTYLES [Sun exposure]
- MODULE 2. HEALTHY LIFESTYLES [Proper nutrition. Practical ideas for maintaining a healthy diet]
- MODULE 2. HEALTHY LIFESTYLES [Alcohol and tobacco consumption. Practical ideas for avoiding consumption]
- MODULE 2. HEALTHY LIFESTYLES [Sleep. Practical ideas for good sleep]
- MODULE 3. WORK [Job stress and Burnout. Training and skills for management]
- MODULE 4. SOCIAL SUPPORT [Social support. Resources and skills]
- TEMPORARY MESSAGES
  - Did you consult them? (YES or NO response)
  - Did you like them? (YES or NO response)

**Multiple-choice question, options (except temporary modules):*

- Didn't consult
- Consulted
- Liked
- Practiced
- Learned something
- Resulted in a positive change for me
- Already knew it beforehand

4. ACCEPTABILITY AND PERCEIVED UTILITY

*** All questions are answered on a scale from 0 to 10 (with 10 being the highest score)*

- Based on your prior experience, do you believe that PsycovidApp is an appropriate tool for the mental health care of healthcare professionals at the frontline of the COVID-19 pandemic?
  - [ANSWER: visual analog scale from 0 (Not at all appropriate) to 10 (Extremely appropriate)]
- Based on your prior experience, how would you rate the amount of information included in the App?
  - [ANSWER: visual analog scale from 0 (Totally inadequate) to 10 (Totally adequate)]

- Based on your prior experience, do you consider PsycovidApp a useful tool for the mental health care of healthcare professionals at the forefront of the COVID-19 pandemic?
    - [ANSWER: visual analog scale from 0 (Not at all useful) to 10 (Extremely useful)]
- Based on your prior experience, how likely are you to recommend PsycovidApp to other healthcare professional colleagues at the forefront of the COVID-19 pandemic?
    - [ANSWER: visual analog scale from 0 (Very unlikely) to 10 (Very likely)]

5. Finally, we would like to know if you have any suggestions for improving the application or any other comments you would like to make. (Open-ended question)
