# Supplementary material for: Process evaluation of PsyCovidApp, a digital tool for mobile devices aimed at protecting the mental health of healthcare professionals during the COVID-19 pandemic: a mixed method study
Source: Front Psychol. 2024 Mar 21;15:1378372. doi: 10.3389/fpsyg.2024.1378372 (PMC10994142; doi:10.3389/fpsyg.2024.1378372)
Supplement: Supplementary file 2 [file Data_Sheet_2.pdf]

Multimedia Appendix 2. Baseline clinical characteristics of participants in the questionnaire and in the intervention group of the PsycovidApp clinical trial.

No significant differences were observed between the two groups regarding the distribution of participants based on the various psychological assessment scales.

		Questionnaire Participants (n=87)	PsycovidApp Intervention Group (n=248)
Age (years) (N, %)			
	Mean (SD)	45.4 (10.1)	42.1 (11.0)
	Median (IQR; range)	46 (38-52; 24-64)	42 (34-51; 22-64)
	<36	17 (19.5)	75 (30.2)
	36 a 45	25 (28.7)	79 (31.9)
	46 a 55	29 (33.3)	60 (24.2)
	>55	16 (18.4)	34 (13.7)
Gender (N, %)			
	Male	9 (10.3)	38 (15.3)
	Female	78 (89.7)	210 (84.7)
Professional category (N, %)			
	Doctor	29 (33.3)	76 (30.6)
	Nurse	26 (29.9)	87 (35.1)
	Nursing Assistant	31 (35.6)	77 (31)
	Others	1 (1.2)	8 (3.2)
Years of Professional Experience (N, %)			
	Mean (DE)	19.2 (8.8)	-
	Median (IQR; range)	20 (13-25; 3-39)	-
	<6	7 (8.1)	-
	6 a 10	8 (9.2)	-
	11 a 20	36 (41.4)	-
	21 a 30	25 (28.7)	-
	> 30	11 (12.6)	-
Workplace (N, %)			
	Primary Care	12 (13.8)	35 (14.1)
	Internal Medicine	12 (13.8)	48 (19.4)
	Intensive Care Unit	12 (13.8)	40 (16.1)
	Hospital Emergencies	7 (8.1)	31 (12.5)
	Home Care	9 (10.3)	19 (7.7)
	Infectious Diseases Unit	6 (6.9)	16 (6.5)
	Others Hospital Units	29 (33.3)	59 (23.8)
Consuming Psychoactive drugs (N, %)			
	No	71 (81.4)	207 (83.5)
	Yes	16 (18.4)	41 (16.5)
Receiving psychotherapy (N, %)			
	No	79 (90.8)	227 (91.5)
	Yes	8 (9.2)	21 (8.5)
Depression, Anxiety & Stress (DASS-21 Global Score)			
	Baseline: mean (SD)	6.0 (4.0)	5.8 (3.9)
	DASS-21 Change (PRE-POST): mean (SD)	1.9 (3.6)	1.9 (3.2)

Depression (DASS-21 subescale)			
	Without symptoms (<5 points)	51 (58.6%)	143 (57.7%)
	Mild (5-6 points)	4 (4.6%)	26 (10.5%)
	Moderate (7-10 points)	21 (24.1%)	55 (22.2%)
	Severe (11-13 points)	6 (6.9%)	15 (6%)
	Extremely severe (>13 points)	5 (5.75%)	9 (3.6%)
Anxiety (DASS-21 subescale)			
	Without symptoms (<4 points)	40 (46.0%)	121 (48.8%)
	Mild (4 points)	11 (12.6%)	32 (12.9%)
	Moderate (5-7 points)	14 (16.1%)	40 (16.1%)
	Severe (8-9 points)	10 (11.5%)	24 (9.7%)
	Extremely severe (>9 points)	12 (13.8%)	31 (12.5%)
Stress (DASS-21 subescale)			
	Without symptoms (<8 points)	34 (39.1%)	104 (41.9%)
	Mild (8-9 points)	10 (11.5%)	28 (11.3%)
	Moderate (10-12 points)	25 (28.7%)	56 (22.6%)
	Severe (13-16 points)	13 (14.9%)	43 (17.3%)
	Extremely severe (>16 points)	5 (5.7%)	17 (6.9%)
Post-Traumatic Stress (DTS)			
	No (<40 points)	51 (58.6%)	150 (60.5%)
	Si (≥40 points)	36 (41.4%)	98 (39.5%)
Burnout (MBI-HSS) Emotional Exhaustion Subscale			
	Low (0-16 points)	33 (37.9%)	95 (38.3%)
	Moderate (17-26 points)	20 (23.0%)	61 (24.6%)
	High (>27 points)	34 (39.1%)	92 (37.1%)
Burnout (MBI-HSS) Personal Accomplishment Subescale			
	High (>39 points)	56 (64.4%)	144 (58.1%)
	Moderate (32-38 points)	18 (20.7%)	65 (26.2%)
	Low (0-31 points)	13 (14.9%)	39 (15.7%)
Burnout (MBI-HSS) Depersonalization Subescale			
	Low (0-6 points)	61 (70.1%)	163 (65.7%)
	Moderate (17-12 points)	16 (18.4%)	50 (20.2%)
	High (>13 points)	10 (11.5%)	35 (14.1%)
Insomnia (ISI)			
	No clinical significance (0-7 points)	38 (46.7%)	102 (41.1%)
	Subclinical Insomnia (8–14 points)	28 (32.2%)	89 (35.9%)
	Moderate clinical insomnia (15–21 points)	18 (20.7%)	49 (19.8%)
	Severe clinical insomnia (22–28 points)	3 (3.4%)	8 (3.2%)
Self-efficacy (GSE)			
	Mean (SD)	32.6/40 (5.1)	32.4/40 (4.7)

DASS-21, Depression, Anxiety, and Stress Scale; DTS, Davidson Trauma Scale; MBI-HSS, Maslach Burnout Inventory - Human Services Survey; GSE, General Self-Efficacy Scale; ISI, Insomnia Severity Index; IQR, Interquartile Range; SD, Standard Deviation
