# Supplementary material for: Process evaluation of PsyCovidApp, a digital tool for mobile devices aimed at protecting the mental health of healthcare professionals during the COVID-19 pandemic: a mixed method study
Source: Front Psychol. 2024 Mar 21;15:1378372. doi: 10.3389/fpsyg.2024.1378372 (PMC10994142; doi:10.3389/fpsyg.2024.1378372)
Supplement: Supplementary file 3 [file Data_Sheet_3.pdf]

Multimedia Appendix 3. *Usage Time of PsycovidApp Reported by Participants in the Post-Intervention Questionnaire.*

Table A3.1. Degree of PsycovidApp use based on participants' age.

TECHNICAL PROBLEMS TO ACCESS	*NO*		*YES*	*P*
N (%)<br>Age (years): mean (SD);<br>[95% IC]	74 (85.1)<br>45 (10.18);<br>[42.64 - 47.36]		8 (9.2)<br>47.25 (7.96);<br>[40.60 - 53.90]	.547
ACCES TO ALL CONTENT				
N (%)<br>Age (years): mean (SD);<br>[95% IC]	15 (17.2)<br>39.27 (9.97);<br>[33.75 - 44.79]		68 (78.2)<br>46.96 (9.45);<br>[44.67 - 49.24]	.006*
USAGE 1st WEEK	*No acces*	*1-3 days/week*	*4-7 days/week*	*P*
N (%)<br>Age (years): mean (SD);<br>[95% IC]	3 (3.4)<br>51.33 (6.11);<br>[36.15-66.51]	23 (26.4)<br>42.96 (9.88)<br>[38.68 -47.22]	57 (65.5)<br>46.24 (9.86);<br>[43.63- 48.86]	.188
USAGE 2nd WEEK				
N (%)<br>Age (years): mean (SD);<br>[95% IC]	5 (5.7)<br>49.2 (7.19);<br>[40.27 - 58.12]	31 (35.6)<br>42.41 (10.84);<br>[38.44 - 46.39]	43 (49.4)<br>47.18 (8.74);<br>[44.49 -49.88]	.077

*CI: Confidence Interval; N: Number of Participants; SD: Standard Deviation.

Tabla A3.2. PsycovidApp Usage Time During the Intervention Based on the Consumption of Psychotropic Drugs

	NO PSYCHOACTIVE DRUGS (N=71)	YES PSYCHOACTIVE DRUGS (N=16)
Daily Use of PsycovidApp (minutes) [a]<br>*N (%)*		
Mean (SD)	22.28 (22.50)	24.37 (8.73)
Median (IQR; range)	15 (10-30;0-120)	25 (15-30;15-45)
0	2 (2.82)	0 (0)
1 a 5	11 (15.49)	0 (0)
6 a 15	28 (39.44)	5 (31.25)

16 a 30	21 (29.58)	10 (62.50)
31 a 60	2 (2.82)	1 (6.25)
> 60	7 (9.86)	0 (0)
Weekly Use of PsycovidApp (hours)[b] *N (%)*		
Mean (SD)	5.35 (5.18)	6.87 (3.07)
Median (IQR; Min-Max)	4 (2-7; 0-24)	6.5 (5.5-8;2-15)
0	2 (2.82)	0 (0)
1-2	22 (30.99)	1 (6.25)
3-5	20 (28.17)	3 (18.75)
6-10	12 (16.90)	11 (68.75)
> 10	7 (9.86)	1 (6.25)
NA	8 (11.27)	0 (0)

IQR, Interquartile Range; N= number of participants; SD, Standard Deviation.

[a]Significant differences between groups (p = 0.043)

[b]Significant differences between groups (p = 0.013).
