# Supplementary material for: Process evaluation of PsyCovidApp, a digital tool for mobile devices aimed at protecting the mental health of healthcare professionals during the COVID-19 pandemic: a mixed method study
Source: Front Psychol. 2024 Mar 21;15:1378372. doi: 10.3389/fpsyg.2024.1378372 (PMC10994142; doi:10.3389/fpsyg.2024.1378372)
Supplement: Supplementary file 4 [file Data_Sheet_4.pdf]

Multimedia Appendix 4. Total number of proposed improvements for PsycovidApp raised by the expert panel classified as appropriate in terms of utility and feasibility

CATEGORY	Utility	Feasibility
**UTILITY**		
1. Add a podcast format for the same written text	8	7
2. Consider it as a health tool, not just as assistance	3	2
3. Delve deeper into techniques and strategies protecting the mental health of healthcare professionals	9	7
4. Prioritize the App's functions concerning the primary need it aims to address	9	7
5. Understand the issue from the user's perspective and avoid falling into stereotypical medical or psychological language	6	7
6. Include gaming elements, gamification, for the app's objectives. Incorporate interactive challenges that can be regularly updated and allow for progressive scaling	8	5
**ATRACTIVENESS**		
7. Accompany the text with more images and videos that explain the content being read (e.g., in the breathing exercises)	5	8
8. Improve visual aspects. UI (User Interface) is neuroscience: More vibrant colours, rounded buttons, clear calls to action, enriching images, highlighted text	8	7
9. Option to change font size	9	9
10. Summary of completed sections or chapters	8	8
11. Add (unlock) content based on goal/subject completion	7	7
12. Use more hierarchically structured, personalized headlines that grab attention. Employ a more conversational language to connect	7	8
13. Add a progress bar for content download during the process	5	8
14. Separate content into 2 sections: notifications/updates and content	7	8
15. Add the option to dismiss notifications without reading them by dragging them laterally	7	7
16. The possibility of entering a work shift/planning and linking mood states to it, as well as advice	7	3
17. The information is very theoretical, change the language used. The positive aspect is that it can be used by professionals in their usual clinical practice with other users	7	8
**EASE OF USE**		
18. Have a content schema available	8	8
19. Enable access to the module from the content guide	8	7
20. Add a video tutorial on how to ideally use the app	6	8
21. Modify navigation with "BACK and NEXT" buttons for direct navigation. Add an easily accessible exit to the main menu without alarming indications but with a more user-friendly and empathetic approach	8	7
22. Quick access to techniques or advice. Display an icon of breathing exercises, for example	6	7
23. Establish a content filter	6	8

24. Direct attention and facilitate decision-making	7	8
25. Modify multimedia content or the system so that: 1) the mobile device does not lock after a few seconds; 2) the multimedia content (e.g., audio recording) continues to play	9	7
26. Add a reminder system or mode for the last completed module/section/topic. Have an overview from the index indicating how many steps are left to view or have been seen in each module. For example: Mark in a different color/shade the modules already consulted	9	8
27. Reduce the amount of information and redesign UI (User Interface) pages for a more user-friendly functionality and better connection with users	7	7
28. Translate warnings or other information into Spanish	4	9
29. The possibility of accessing pages in Spanish, talks in Spanish adapted (e.g., sleep)	7	9
**WIDESPREAD USAGE ON A LARGE SCALE**		
30. Need to break stigma	6	5
31. Direct it to another segment of the population	8	6
32. Adapt content to a "non-covid" context	9	9
33. Improve interoperability	7	5
34. Specify where they can ask for help (mental health/physical activity/nutrition)	8	8
35. Resilience is explained in one of the modules but appears in the module presentation as if it were a familiar concept	7	7
**ADDING CONTENT**		
36. Offer different difficulty levels: write modules on the same topic but at a more advanced level	9	7
37. Also create some simpler content, especially in emotional regulation	8	8
38. Possibility of including a scale (anxiety, Maslach Burnout…)	8	7
39. Add forms or a self-assessment system	9	7
40. Add explanations or clarify some technical words used. Example: verbiage	8	9
41. In some modules like possible stress reactions, it might be interesting for the user to mark identified reactions as a self-awareness process	9	7
42. In relaxation/exercise sections, offer a link to a video with basic relaxation exercises with 2-3 difficulty levels. Example: (1st level) - exercises in bed or on a mat based on diaphragmatic breathing, simple mobility exercises, and background music	7	6
43. Add a short voluntary questionnaire of perhaps 2-3 True/False questions at the end of each module. / Add a brief summary of the most important content of the module	7	8
44. Add a module about pain	7	7
45. Add notifications at certain times of the day	7	7
**REMOVING CONTENT**		
46. Modules M2.1 and M2.2 seem somewhat redundant; perhaps it could be condensed into one.	7	9
47. Remove content related to the study (if it has finished)	7	9

48. Remove and update invalid links (e.g., the link from the Ministry of Health, Consumption, and Social Welfare in module 2.2. in section 11/13 in "Practical Ideas for Physical Exercise")	8	9
**FEEDBACK**		
49. Automatic (and if possible personalized) feedback	9	7
50. Create a support community	8	5
51. Access specialized professionals for psychological assistance to healthcare personnel	6	6
52. Add social support by connecting with users in similar situations	8	4
53. Automate the evaluation process	7	7
54. Add a link to terms and conditions and the privacy policy in "Accept Terms and Conditions and the Privacy Policies"	5	7
55. Update the data protection policy accurately and specifically for the App	7	8
56. Remove geolocation from the App	8	8
57. Ensure technical stability for the App across different systems or devices	9	6
